# Temperature induced crossing in the optical bandgap of mono and bilayer MoS_2_ on SiO_2_

**DOI:** 10.1038/s41598-018-23788-3

**Published:** 2018-03-29

**Authors:** Youngsin Park, Christopher C. S. Chan, Robert A. Taylor, Yongchul Kim, Nammee Kim, Yongcheol Jo, Seung W. Lee, Woochul Yang, Hyunsik Im, Geunsik Lee

**Affiliations:** 1 Department of Chemistry, School of Natural Science, Ulsan National Institute of Science and Technology (UNIST), Ulsan, 44919 Korea; 2 Clarendon Laboratory, Department of Physics, University of Oxford, Oxford, OX1 3PU UK; 3 Department of Physics, Soongsil University, Seoul, 06978 Korea; 4 Division of Physics and Semiconductor Science, Dongguk University, Seoul, 04620 Korea; 5 Department of Physics, Hong Kong University of Science and Technology, Clear Water Bay, Hong Kong, China

## Abstract

Photoluminescence measurements in mono- and bilayer-MoS_2_ on SiO_2_ were undertaken to determine the thermal effect of the MoS_2_/SiO_2_ interface on the optical bandgap. The energy and intensity of the photoluminescence from monolayer MoS_2_ were lower and weaker than those from bilayer MoS_2_ at low temperatures, whilst the opposite was true at high temperatures above 200 K. Density functional theory calculations suggest that the observed optical bandgap crossover is caused by a weaker substrate coupling to the bilayer than to the monolayer.

## Introduction

The discovery of unique transport properties of graphene prepared by mechanical exfoliation has spurred many new research activities for future electronic devices because of graphene’s intriguing energy band structure and high carrier mobility^1–3^. Although graphene is a promising material due to its rich physics, pristine graphene has no bandgap which can limit application areas. As alternatives, layered two-dimensional (2D) materials composed of transition metal dichalcogenides (TMDs) such as MX_2_ (M = Mo, W, and X = S, Se) have been the centre of attention for applications in next-generation nanoelectronic and optoelectronic devices because of their unusual valley and optical polarization properties. Amongst them, MoS_2_ can provide both indirect and direct bandgap transitions depending on the layer thickness^4–6^. A monolayer (1 L) of MoS_2_ (1L-MoS_2_) is a direct gap semiconductor with a band gap of 1.8~1.9 eV at the K-points of the 2D hexagonal Brillouin zone, whereas bulk MoS_2_ is an indirect semiconductor with a band gap of ~1.2 eV^4–6^. These findings have boosted the development of 2D materials for high-performance flexible electronic and optoelectronic devices^7,8^. There has been much interest generated in studying the characteristic optical properties of MoS_2_ using photoluminescence (PL) measurements as well as the valleytronics related to its 2D symmetry^9–15^. However, the electrical and optical properties of the MoS_2_ can be greatly affected by its surface and also by the MoS_2_/substrate interface. It is therefore important to understand how such interfaces can affect the optical and electronic features of the material. Moreover, the PL intensity depends on the number of layers, indicating that the quantum efficiency can decrease with layer thickness and whether the flake is freestanding or on a substrate^6^. Note that when MoS_2_ layers lie on a substrate, each layer undergoes a different strain between the substrate and the MoS_2_ layers because the first layer of MoS_2_ is in direct contact with the substrate, whilst the other layers interact weakly due to van der Waals bonding between the MoS_2_ layers, which can affect the optical transition between the 1L-MoS_2_ and the other layers.

In this letter, we demonstrate temperature dependent PL behaviour of mechanically-exfoliated 1L- and bilayer (2 L) MoS_2_ prepared on a SiO_2_ substrate. The PL peak’s intensity and energy for the 2L-MoS_2_ are stronger and higher than those of the 1L-MoS_2_ at low temperatures below 200 K, in contrast to the room temperature measurements, where the opposite occurs. In order to explain this phenomenon, density functional theory (DFT) calculations are performed taking into account the thermal expansion at the MoS_2_/SiO_2_ interface.

## Results

**Figure 1:**
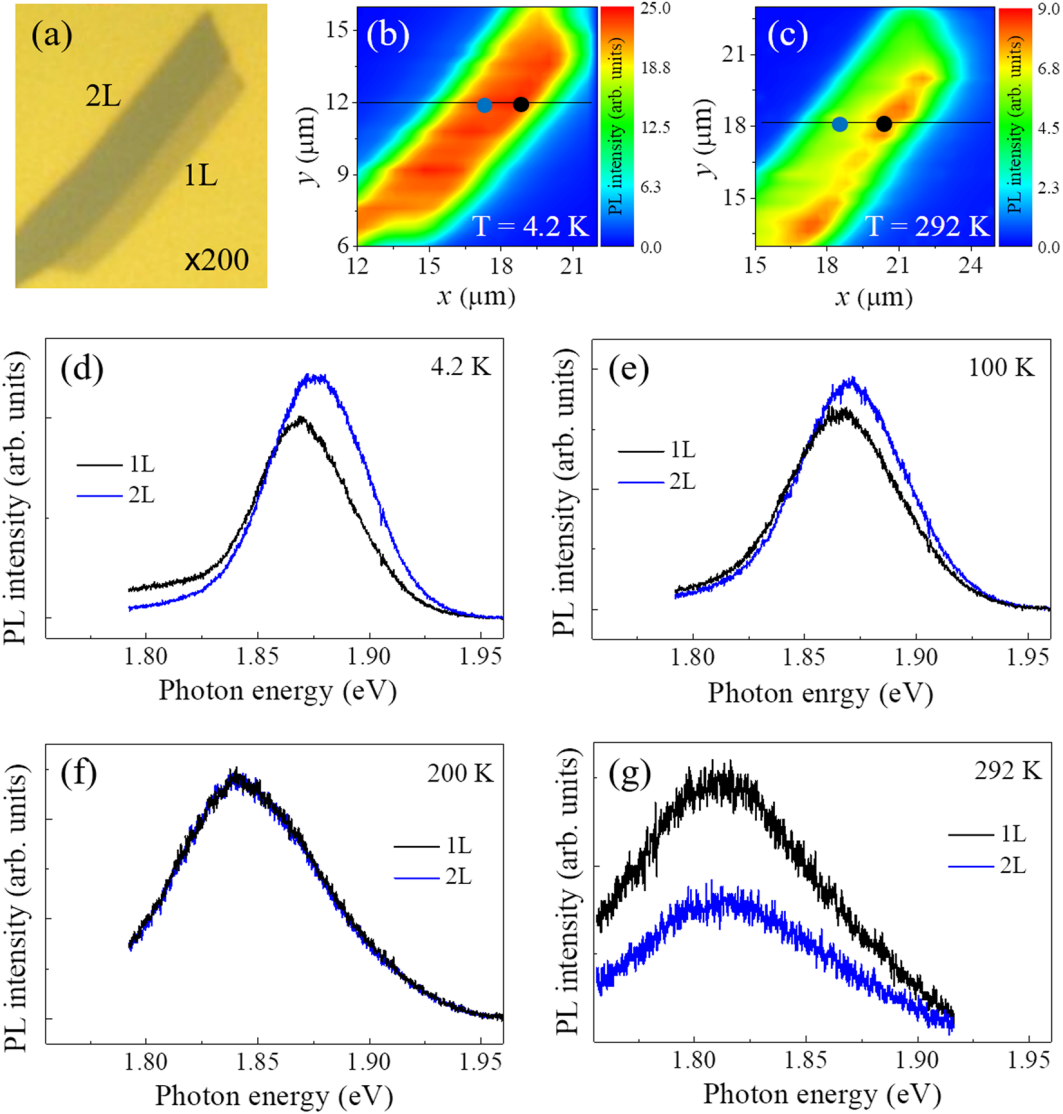
(**a**) Optical microscope image of the MoS_2_ flake prepared by mechanical exfoliation of MoS_2_ placing on a SiO_2_ substrate. The semi-transparent and dark regions of the MoS_2_ flake correspond to 1L- and 2L-MoS_2_, respectively. μ-PL spectral maps of 1L- and 2L-MoS_2_ flake measured at 4.2 K (**b**) and 292 K (**c**). An excitation power of 1 mW/cm^2^ is used. Here, *x* and *y* are arbitrary direction on the microscope image. μ-PL spectra of 1L- and 2L-MoS_2_ flake extracted from the circle points of the maps at 4.2 K (**d**), 100 K (**e**), 200 K (**f**), and 292 K (**g**), respectively.

**Figure 2:**
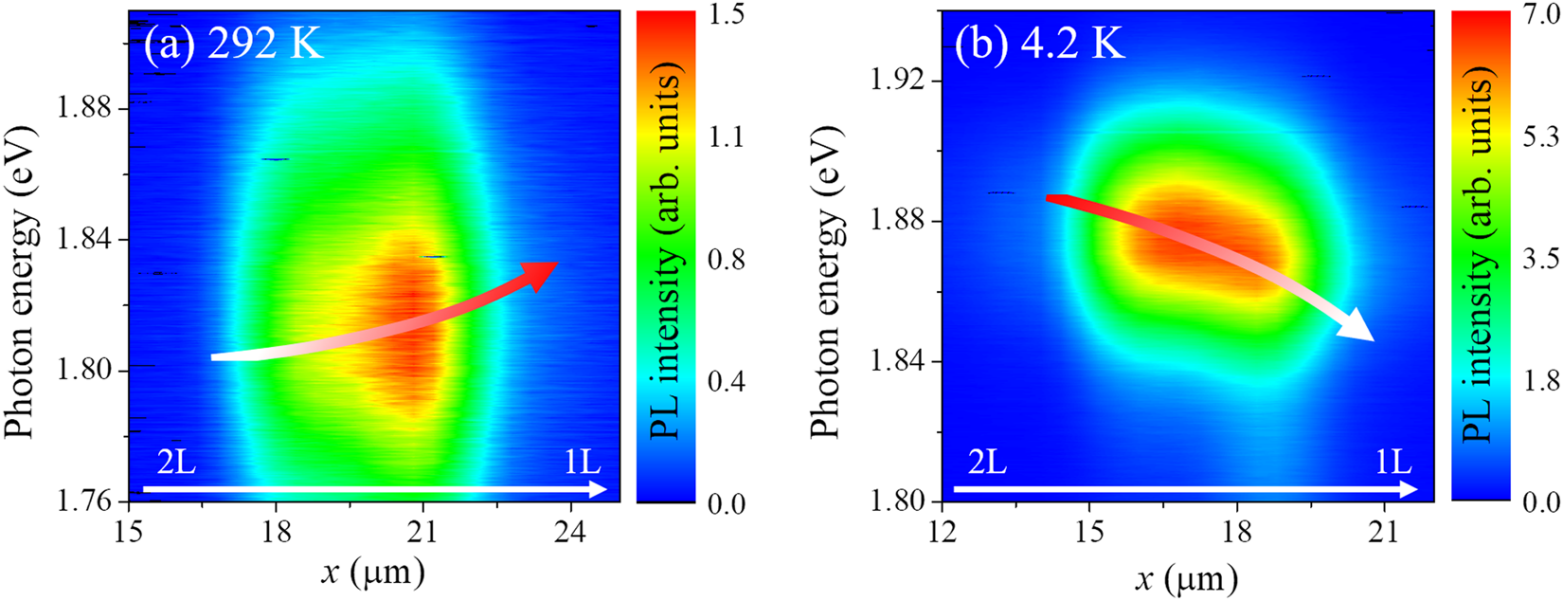
μ-PL spectra maps at 292 K (**a**) and 4.2 K (**b**), measured along the dotted lines in the intensity maps of Fig. 1(b,c). The red arrows are the guide for eyes indicating the PL peak’s energy and intensity variation from 2L- to 1L-MoS_2_.

Figure 1(b,c) show the PL spectral maps measured at the 1L- and 2L-MoS_2_ flake which are consistent with the optical microscopy image in Fig. 1(a). Figure 1(d~g) show the PL spectra of the 1L- and 2L-MoS_2_ flakes, extracted from the circle points of the maps measured at 4.2 K, 100 K, 200 K and 292 K, respectively. The PL intensity measured at 4.2 K is similar across both the 1L- and 2L-MoS_2_ regions. However, by inspecting the PL spectra taken at 4.2 K along the dotted line on the 1L- and 2L-MoS_2_ as shown in Fig. 1(d), the intensity of the 2L-MoS_2_ is slightly more intense than that of 1L-MoS_2_. In addition, a prominent PL peak can be identified at a higher energy of ~1.869 eV for 1L-MoS_2_ than 1.876 eV for 2L-MoS_2_ at 4.2 K, while the opposite is true at room temperature as shown in Fig. 1(g). We have checked the variation of PL intensity and emission energy for the 1L- and 2L-MoS_2_ at the several temperatures. The PL peak’s intensity and energy for the 2L-MoS_2_ are more intense and higher than those of the 1L-MoS_2_ up to 150 K, whilst the energies are almost the same near 200 K (Fig. 2(f)) and then become inverted at room temperature, which is consistent with previous reports^6^. The boundary between the 1L- and 2L-MoS_2_ regions indicated in the optical microscope image can be seen clearly. The abrupt PL intensity difference between 1L- and 2L-MoS_2_ at 292 K coincides with the corresponding regions of the optical microscopy image, confirming the difference in PL collected from the two distinct areas of the flake. The observed room-temperature PL behavior of the 1L- and 2L-MoS_2_ is in contrast to the previous reports, stating that the PL intensity of the MoS_2_ decreases with increasing layer thickness^6^. We should consider the relationship of the emission energy and the physical nature of the MoS_2_/SiO_2_ interface, which may cause different strain in 1L- and 2L-MoS_2_. Mechanical strain can reduce the optical band gap by ~45 meV/% for monolayer MoS_2_ and ~120 meV/% for bilayer MoS_2_^16^, where the role of substrate is unclear. Similar PL characteristics were observed in another flake on the same SiO_2_ substrate, confirming that this phenomenon is reproducible (Fig. S1)^17^.

The detailed PL intensity variations across the flake (dotted lines of Fig. 1(b,c)) are presented in Fig. 2. The PL intensity and energy at 4.2 K becomes slightly weaker and redshifts across the 2L-MoS_2_ to 1L-MoS_2_ boundaries as shown in Fig. 2(b). However, the opposite behavior is seen above 200 K, and the signal becomes more intense with increasing *x* position, as shown in Fig. 2(a).

**Figure 3:**
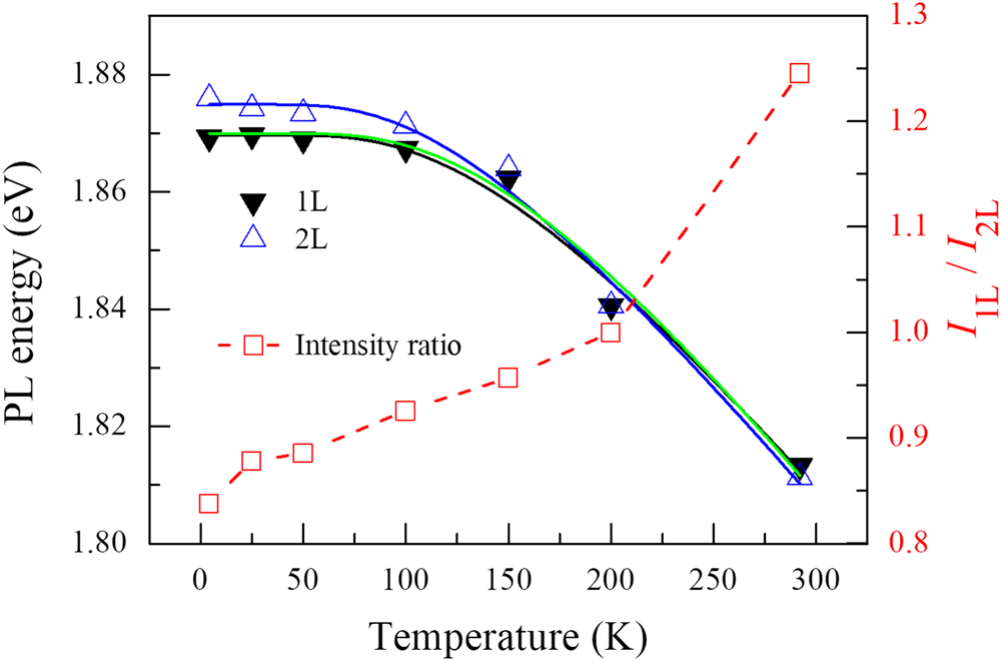
PL energy and integrated PL intensity ratio of the 1L- and 2L-MoS_2_ as a function of temperature. The solid lines are fitting curves using the O’Donnell and Chen equation.

Figure 3 shows the PL peak energies for the 1 L and 2L-MoS_2_ and their intensity ratio (I_1L_/I_2L_) as a function of temperature. The PL peaks shift to lower energy with increasing temperature. The temperature-dependent optical bandgap variation is understood in terms of lattice dilation and electron-lattice interactions. We can clearly see that the peak energy crosses over around 200 K. In addition, the intensity of the 1L-MoS_2_ becomes more intense in the high temperature region above ~200 K. The temperature dependence of the bandgap proposed by O’Donnell and Chen takes into account the influence of phonons on the bandgap energy to obtain a better fit for semiconductors at lower temperatures^18^. They considered the following equation: ${{\rm{E}}}_{{\rm{g}}}({\rm{T}})={{\rm{E}}}_{{\rm{g}}}(0)-{\rm{S}}\langle {{\rm{E}}}_{{\rm{ph}}}\rangle [\coth (\langle {{\rm{E}}}_{{\rm{ph}}}\rangle /2{{\rm{k}}}_{{\rm{B}}}{\rm{T}})-1]$, where $\langle {{\rm{E}}}_{{\rm{ph}}}\rangle$ is an average phonon energy and *S* is a dimensionless coupling constant. The measured data are in good agreement with the aforementioned relationship at all measured temperatures (black solid line for 1L-MoS_2_ and blue solid line for 2L-MoS_2_). The extracted $\langle {{\rm{E}}}_{{\rm{ph}}}\rangle$ value is ~37.8 meV for the 1L-MoS_2_ and 33.1 meV for the 2L-MoS_2_. The theoretical LO inter-valley phonon energy of the 1L-MoS_2_ is 41 meV^19^, which is close to our fitted value. The green line is the fitting curve for the 1L-MoS_2_ with <E_ph_> = 41 meV.

## Discussion

In order to explain the observed crossing behavior of the optical bandgap of the 1L- and 2L-MoS_2_ with increasing temperature, DFT calculations were carried out focusing on the effect of the substrate. The effect of electron-phonon coupling on the energy gap can be recast in terms of the lattice thermal expansion as an equivalent phenomenological description^18^. Furthermore, the interface electronic coupling should be nearly independent of temperature, because the band edge states at the K or K’ valley mainly arise from Mo *d* orbitals with a negligible coupling to the O or Si atomic orbitals of the substrate. The band alignment is type I with a much wider energy gap for SiO_2_ than MoS_2_^20,21^. Thus, the temperature dependence of the energy gap can be understood by the thermal expansion of the 1L- or 2L-MoS_2_ themselves. For example, the lowest MoS_2_ layer will have a different coupling strength to the underlying substrate for 1L- MoS_2_ due to an additional van der Waals attraction in bilayer MoS_2_, thus the in-plane thermal expansion can be different for 1L- and 2L-MoS_2_.

The relative coupling strength is studied by calculating the binding energy between 1L-MoS_2_ and SiO_2_, and comparing that to the interlayer binding energy of 2L-MoS_2_, where the Perdew-Burke-Ernzerhof (PBE)^22^ type generalized gradient approximation is used together with the D2 method^23^ for the van der Waals (vdW) correction. The binding energy between 1L-MoS_2_ and SiO_2_ is calculated by ${E}_{b}^{1{L-\text{MoS}}_{2}/{{\rm{SiO}}}_{2}}={E}_{1{L \mbox{-} \text{MoS}}_{2}/{{\rm{SiO}}}_{2}}-{E}_{1{L \mbox{-} \text{MoS}}_{2}}-{E}_{{{\rm{SiO}}}_{2}}$, where ${E}_{1{L \mbox{-} \text{MoS}}_{2}/{{\rm{SiO}}}_{2}}$ is the total energy of the adsorbed system, ${E}_{1{\rm{L}}-{{\rm{M}}{\rm{o}}{\rm{S}}}_{2}}$ and ${E}_{{{\rm{SiO}}}_{2}}$ are the energies of 1L-MoS_2_ and SiO_2_, respectively, for its own optimized lattice constant. The calculated binding energy is −139 meV per MoS_2_ unit, in good agreement with theoretical reports^20,21^, indicating that adsorption of 1L-MoS_2_ on SiO_2_ is energetically favorable. However, the interlayer binding energy of 2L-MoS_2_ is calculated to be −146 meV per MoS_2_ unit, which is 7 meV/MoS_2_ more stable than the adsorption of the monolayer. We note that using the Tkatchenko and Scheffler type vdW correction^24^ gives qualitatively the same result (304 meV for MoS_2_-MoS_2_ versus 235 meV MoS_2_-SiO_2_). Thus, the bottom MoS_2_ layer in 2L-MoS_2_ should be less coupled to the SiO_2_ than the upper layer, giving rise to almost free-standing 2L-MoS_2_.

The average phonon energy <E_ph_> extracted from the fitting in Fig. 3 is closer to the energy of the in-plane phonon mode (E_2g_) than that of the out-of-plane mode (A_1g_), which means that the in-plane thermal expansion can be used to understand the observed PL gap behavior. Since the 1L-MoS_2_ has a stronger coupling to the SiO_2_ substrate than 2L-MoS_2_ according our theoretical calculation, the in plane thermal expansion of 1L-MoS_2_ should be suppressed compared to that of 2L-MoS_2_, due to a larger contribution from the lighter elements (Si and O) in the vibration, where the thermal expansion coefficient (TEC) of SiO_2_ is ~10^−6^/K, and the in plane TEC of bulk MoS_2_ was measured to be ~5.0 × 10^−6^/K^25^, and a monolayer of MoS_2_ has a much larger value ~24.4 × 10^−6^/K from a recent measurement^26^. Thus, a smaller expansion of 1L-MoS_2_ than 2L-MoS_2_ arises when the thermal expansion is modified by the substrate coupling. Then as the lattice parameters increase, the direct energy gap variation can be used to explain the observed temperature dependence of the gap, as long as the substrate electronic coupling is independent of temperature and the phenomenological description of ref.^18^ for the electron-phonon coupling via lattice thermal expansion holds true for our system.

**Figure 4:**
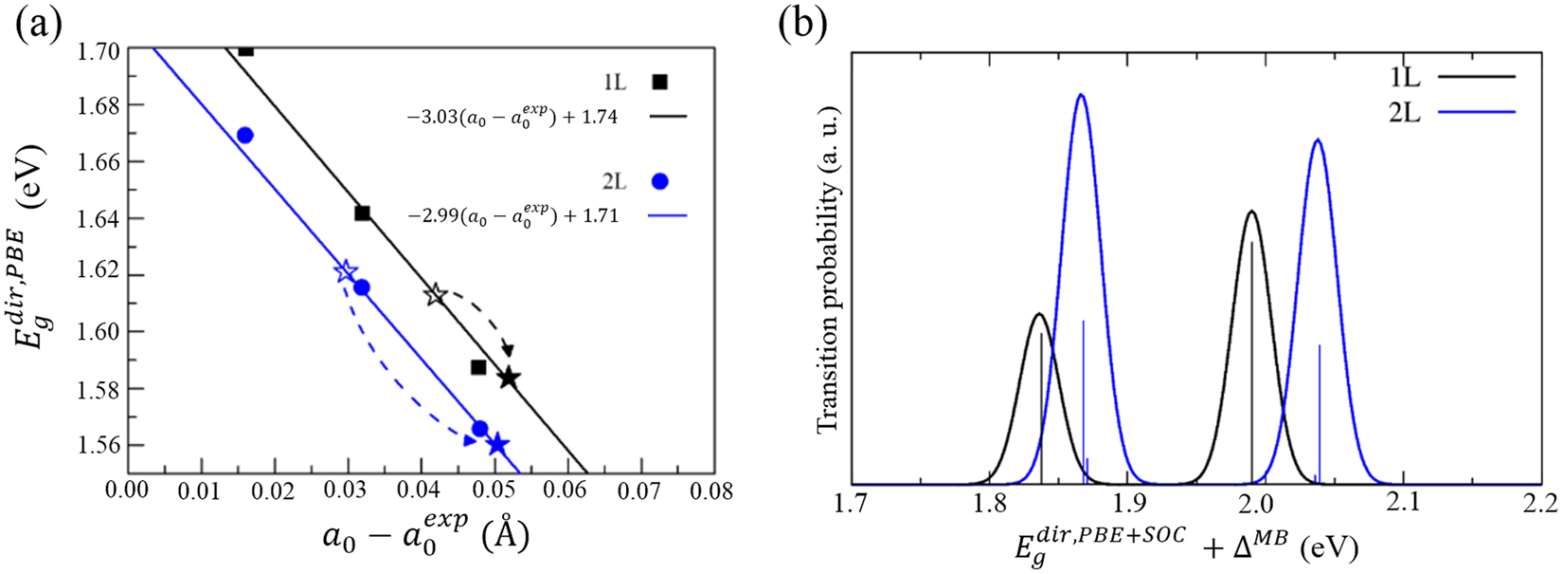
(**a**) Calculated direct energy gap $({E}_{g}^{\text{dir},\text{PBE}})$ variation of the 1L- and 2L- MoS_2_ upon lattice expansion with respect to the experimental value ${a}_{0}^{\exp }=3.16\,\AA$, where the lines are the least-square fit to the results. Note that the dashed arrows represent the redshift of the gap upon thermal expansion by a temperature increase of 300 K, where 2L-MoS_2_ is assumed free-standing and 1L-MoS_2_ experiences substrate strain. The ground state configuration (open stars) is chosen as the optimal lattice constant (*a*_0_ = 3.19 Å) of the free-standing bilayer for 2L-MoS_2_, and arbitrarily for 1L-MoS_2_ to be consistent with the PL data. (**b**) Calculated dipole transition probability of 1 L and 2 L MoS_2_ at the level of PBE including the spin orbit coupling (SOC) with the many body correction Δ^MB^ = 0.3 eV. Vertical lines are the raw intensities rescale for visibility, and they are broadened by Gaussian functions with a FWHM = 0.02 eV.

Figure 4(a) shows the direct gaps calculated for free-standing 1L- and 2L-MoS_2_, indicated by the filled squares and circles, respectively, where the PBE type functional used is sufficient for qualitative analysis. We have chosen certain lattice constants larger than the experimental value ${a}_{0}^{\exp }=3.16\,\AA$. Each of 1L- and 2L-MoS_2_ exhibits a linear variation with an almost equal slope (~ −3.0 eV/Å), where only three data points are displayed in Fig. 4(a) among ten points from 0.0 to 0.16 Å expansion relative to ${a}_{0}^{\exp }$. By using the reported TEC ~2 × 10^−5^/K^27^, we obtain ~ 0.02 Å lattice expansion with a temperature increase of 300 K, thereby predict 60 meV decrease in the gap, as illustrated by the blue dashed arrow in Fig. 4(a). Assuming a full strain of 2L-MoS_2_, it matches well to our experimental result. To explain the crossover behavior, however, we have to choose a suitable size of the gap for 1L-MoS_2_ at low temperature, which is smaller than that of 2L-MoS_2_ as observed in the experiment. Actually, our calculated energy gap with including the spin orbit coupling shows larger gap for 2L-MoS_2_ than 1L-MoS_2_, as shown in Fig. 4(b). With a smaller expansion for 1L-MoS_2_, (black dashed arrow in Fig. 4(a) corresponds to half of that for 2L-MoS_2_), a crossing behavior of the 1L- and 2L-MoS_2_ energy gaps upon thermal expansion can be obtained. Also Fig. 4(b) shows that the intensity is larger for 2L-MoS_2_ than 1L-MoS_2_, which matches with the low temperature observation. This is because the degeneracy is doubled in 2L-MoS_2_ by the presence of the inversion symmetry. The crossing behavior of PL intensity with increasing temperature can be described qualitatively by the enhanced phonon coupling towards the indirect emission in the indirect gap 2L-MoS_2_, thereby the original direct emission will be suppressed in 2L-MoS_2_ whilst it is essentially unaffected in the direct gap 1L-MoS_2_.

In summary, we have demonstrated an abnormal behaviour in the excitonic photoluminescence of 1L- and 2L-MoS_2_. The PL peak’s energy and intensity of monolayer MoS_2_ are lower and weaker compared to bilayer MoS_2_ at low temperatures, whilst the opposite is true at high temperatures above 200 K. The DFT calculations suggest that the observed crossing of the optical bandgap with increasing temperature is due to different thermal expansion coefficients of the 1L- and 2L-MoS_2_ at the MoS_2_/SiO_2_ interface, causing weaker substrate coupling to the bilayer than to the monolayer, which enhances the redshift of the bilayer MoS_2_.

## Methods

### Preparation and characterization of 1L- and 2L-MoS_2_

The 1L- and 2L-MoS_2_ flakes were prepared on a SiO_2_/Si substrate by mechanical exfoliation from natural MoS_2_ as shown in Fig. 1(a) and the number of MoS_2_ layers was characterized by micro-Raman spectroscopy that the frequency differences between the in-plane (E^1^_2g_) and out-of-plane (A_1g_) modes are 17.77 cm^−1^ for the 1L-MoS and 20.01 cm^−1^ for the 2L-MoS_2_ (Fig. S2), which are in good agreement with values in the literature^28^. For the low temperature PL measurements, the sample was mounted in a continuous-flow helium cryostat, allowing the temperature to be controlled accurately from low temperature (4.2 K) to room temperature and the optical luminescence properties were characterized by using micro-photoluminescence (μ-PL). A CW linearly polarized solid-state laser operating at a wavelength of 532 nm was used for the excitation of the MoS_2_ flake. A 100× (NA 0.7) objective was held above the cryostat focusing the incident laser beam to a spot size of ~0.8 μm^2^ and also to collect the emitted luminescence from the same spot.

## Calculations

The atomic structure is modelled as a slab, which includes six atomic layers and 20 Å vacuum space, and bottom Si dangling bonds are passivated by H (Fig. S3). It should be noted that the substrate involves O dangling bonds at the surface and the surface O atoms are reconstructed, which is consistent with a previous theoretical report^20^. The interface between the SiO_2_ and the MoS_2_ layer is calculated by choosing the lateral supercell size as 9.69 Å matching 3 × 3-MoS_2_ and 2 × 2-SiO_2_ to minimize the lattice mismatch to as small as 1.2%, where our optimized lattice constants for pristine MoS_2_ and SiO_2_ are *a*_0_ = 3.19 Å (comparable to the experimental value ${a}_{0}^{\exp }$ = 3.16 Å) and 4.90 Å (experimental value is 4.91 Å), respectively. The optimized vertical distance between the lowest S and the highest O is 3.13 Å, which indicates a weak interface bond of the van der Waals type. It was also shown that other termination types show a weak interaction of similar magnitude^21^.

## Electronic supplementary material

Supplementary Information
