# Supplementary material for: Temperature induced crossing in the optical bandgap of mono and bilayer MoS2 on SiO2
Source: Sci Rep. 2018 Mar 29;8:5380. doi: 10.1038/s41598-018-23788-3 (PMC5876333; doi:10.1038/s41598-018-23788-3)
Supplement: Supplementary file 1 — Supplementary Information [file 41598_2018_23788_MOESM1_ESM.docx]

*Supplementary Information on*

Temperature induced crossing in the optical bandgap of mono and bilayer MoS_2_ on SiO_2_

Youngsin Park^1^, Christopher C. S. Chan^2,5^, Robert A. Taylor^2,*^, Yongchul Kim^1^, Nammee Kim^3^, Yongcheol Jo^4^, Seung W. Lee^4^, Woochul Yang^4^, Hyunsik Im^4,**^, and Geunsik Lee^1,***^

*^1^Department of Chemistry, School of Natural Science, Ulsan National Institute of Science and Technology (UNIST), Ulsan 44919, Korea*

*^2^Clarendon Laboratory, Department of Physics, University of Oxford, Oxford, OX1 3PU, UK.*

*^3^Department of Physics, Soongsil University, Seoul 06978, Korea*

*^4^Division of Physics and Semiconductor Science, Dongguk University, Seoul 04620, Korea*

*^5^Department of Physics, Hong Kong University of Science and Technology, Clear Water Bay, Hong Kong, China*

*robert.talyor@physics.ox.ac.uk, **hyunsik7@dongguk.edu, [***gslee@unist.ac.kr](mailto:***gslee@unist.ac.kr)


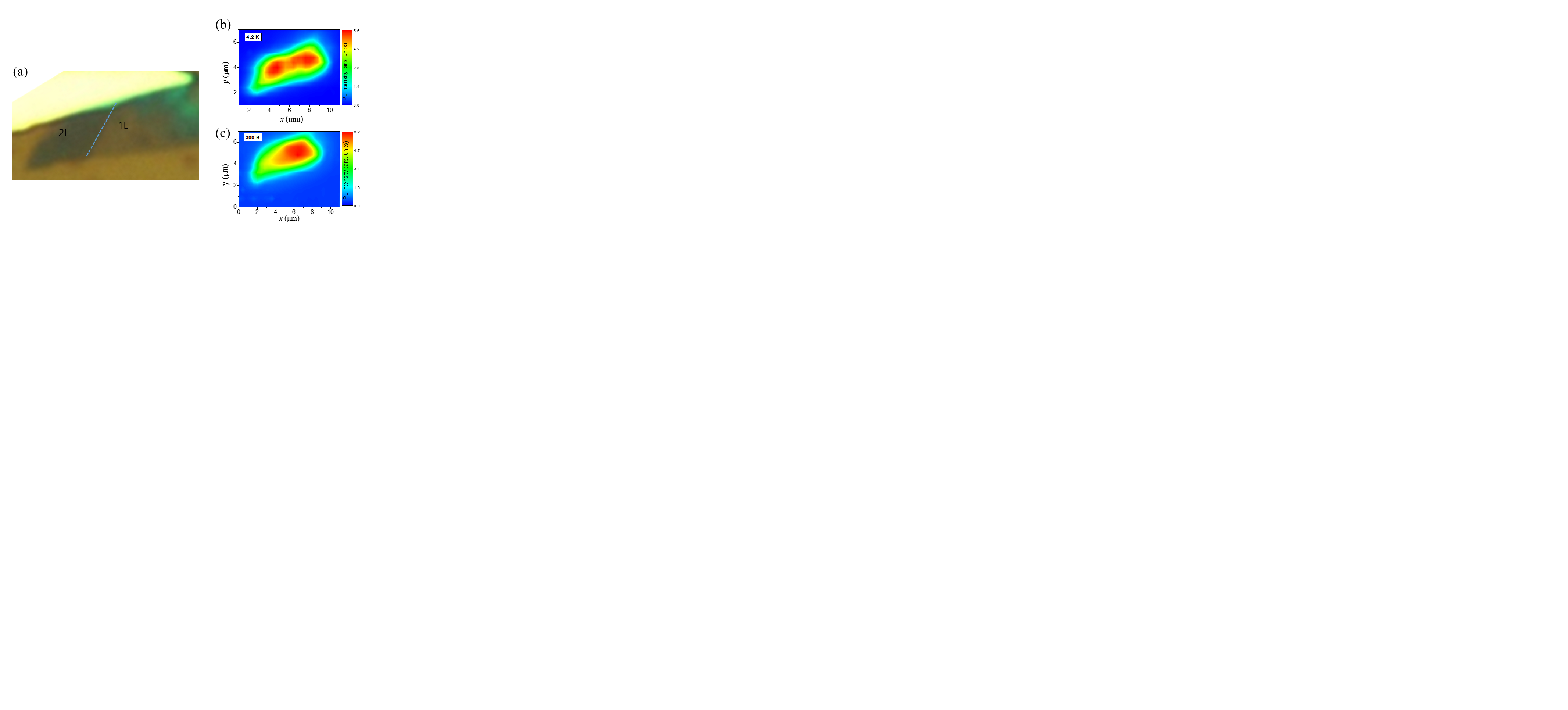


**Figure S1.** (a) Optical microscopy image of another MoS_2_ flake on a SiO_2_ substrate prepared by mechanical exfoliation. The semi-transparent and dark regions of the MoS_2_ flake correspond to 1L- and 2L-MoS_2_, respectively. Micro PL mapping of the 1L- and 2L-MoS_2_ measured at 4.2 K (b) and 292 K (c). The intensity of the 2L-MoS_2_ is slightly higher than that of 1L-MoS_2_, which is similar to that in Fig. 1, while the opposite is true at room temperature (292 K).


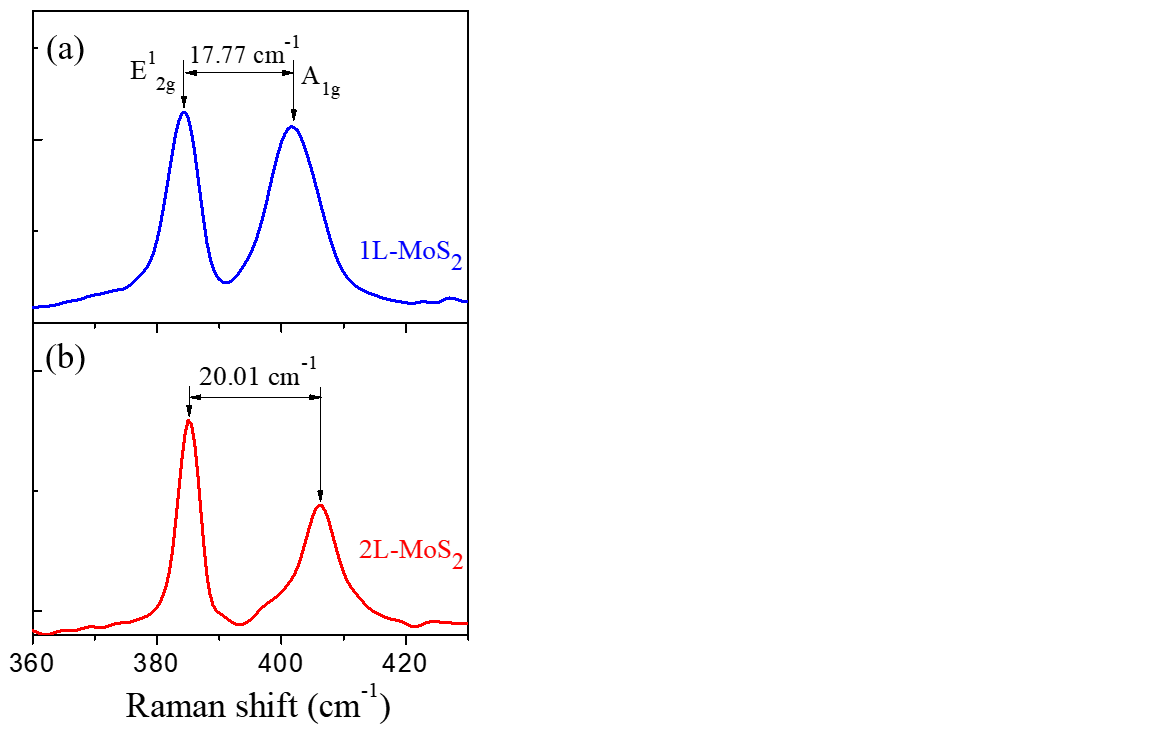


**Figure S2**. Raman spectra taken at the same point as the measured PL of the MoS_2_ flake at room temperature. (a) 1L MoS_2_ and (b) 2L-MoS_2_.


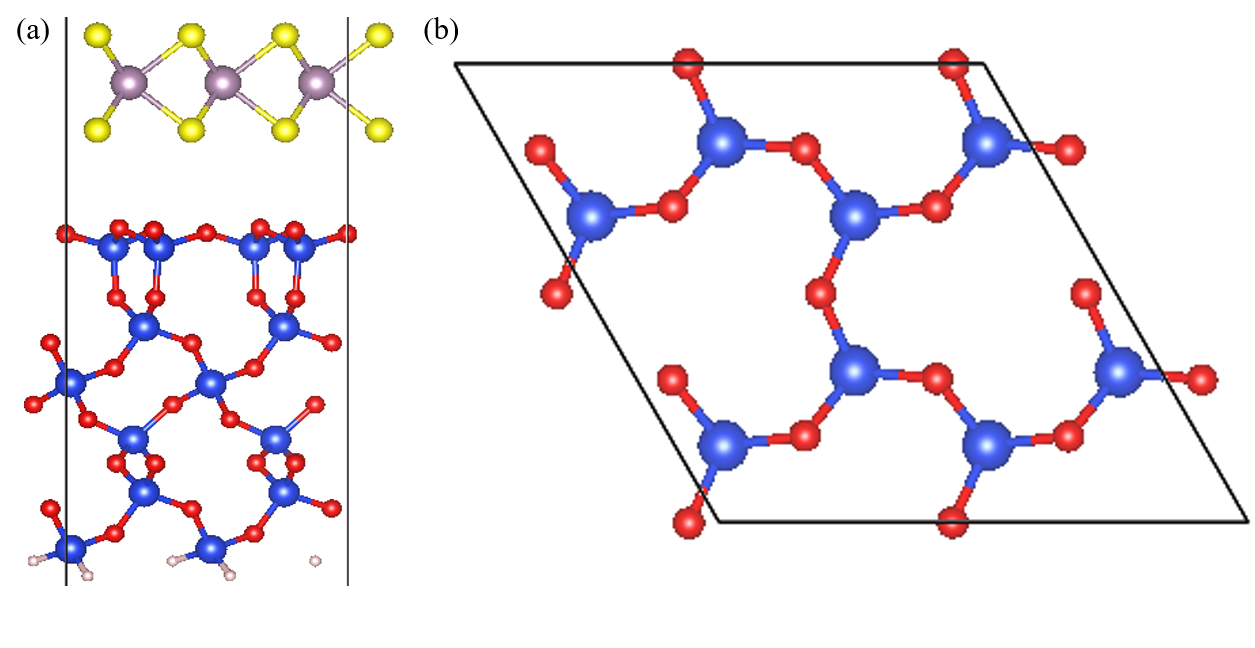


Figure S3. (a) Schematic model of 1L-MoS_2_/SiO_2_ in side view, (b) Schematic view of the top layer of SiO_2_.
